# Safety and Effectiveness of ERCP in the Pediatric Population: A Tertiary Care Center Experience

**DOI:** 10.3390/children13081073

**Published:** 2026-08-13

**Authors:** Dario Ligresti, Dario Quintini, Lucio Carrozza, Gabriele Rancatore, Margherita Pizzicannella, Marco Giacchetto, Maria Vittoria Grassini, Giacomo Emanuele Maria Rizzo, Gaetano Burgio, Gennaro Martucci, Giusy Ranucci, Davide Cintorino, Fabio Tuzzolino, Mario Traina, Ilaria Tarantino

**Affiliations:** 1 IRCCS ISMETT, 90127 Palermo, Italy; dquintini@ismett.edu (D.Q.); luciocarrozza@aopapardo.it (L.C.); grancatore@ismett.edu (G.R.); mpizzicannella@ismett.edu (M.P.); cmgiacchetto@ismett.edu (M.G.); mvgrassini@ismett.edu (M.V.G.); gburgio@ismett.edu (G.B.); gmartucci@ismett.edu (G.M.); granucci@ismett.edu (G.R.); dcintorino@ismett.edu (D.C.); ftuzzolino@ismett.edu (F.T.); mtraina@ismett.edu (M.T.); itarantino@ismett.edu (I.T.); 2 UPMC Italy, 90133 Palermo, Italy; 3 Surgical and Critical Area (MePreCC), Department of Precision Medicine in Medical, University of Palermo, 90133 Palermo, Italy

**Keywords:** ERCP, pediatric, biliopancreatic

## Abstract

**Highlights:**

**What are the main findings?**

- Pediatric ERCP achieved high technical success (91% in patients < 10 Kg and 96% in patients > 10 Kg).
- Among first-time ERCPs, pancreatic indications were associated with a higher rate of adverse events than biliary indications (30% vs. 4.7%; OR 8.28, exact 95% CI 0.93–74.90).

**What are the implications of the main findings?**

- ERCP can be performed effectively and with a low rate of peri-procedural adverse events in infants under 10 kg, in centers combining high procedural volume, experienced biliopancreatic endoscopists, dedicated pediatric anesthesia and multidisciplinary support.

**Abstract:**

**Background and Aim**: Pediatric ERCP remains significantly less studied compared to its application in adults, particularly in infants, due to the limited procedural volume. This study aims to evaluate the indications, procedural specifics, technical success rates, and adverse events associated with pediatric ERCP at a tertiary care center over an 18-year period, with specific attention to patients weighing less than 10 kg. **Methods**: A retrospective analysis of all ERCPs performed on patients under 18 years of age at ISMETT between 2005 and 2023. Demographic data, indications, procedural specifics, outcomes, and adverse events were collected and reported. Subgroup analyses focusing on initial ERCP procedures were performed and stratified by weight and indication type. **Results**: 194 ERCPs were performed on 84 patients. Acute or chronic pancreatitis was the most common indication. Technical success was high across weight groups. Adverse events occurred only in patients ≥ 10 kg (7%), predominantly post-ERCP pancreatitis. No adverse events were reported in the <10 kg group. In the “first-ERCP” subgroup, pancreatic indications were associated with a higher adverse event rate than biliary indications, although the estimate was imprecise owing to the small number of events. **Conclusions**: In our experience as a specialized center, pediatric ERCPs have proven to be safe and effective, even in infants < 10 kg. Pancreatic indications appeared to carry a higher risk for adverse events than biliary indications, although this estimate was imprecise.

## 1. Introduction

Endoscopic retrograde cholangiopancreatography (ERCP) is an endoscopic technique combining gastrointestinal endoscopy and fluoroscopy for the diagnosis and treatment of pancreatic and biliary diseases. Its diagnostic and therapeutic role in adults is well established, with nearly 60 years of practice and robust safety data. In contrast, experience with ERCP in pediatric populations, especially infants, remains limited due to the lower incidence of biliopancreatic diseases and the consequent reduced caseload. Therefore, such procedures are generally referred to specialized hub centers to ensure greater expertise, more comprehensive data collection, and cost efficiency. Sharing equipment and accessories with busy adult endoscopy units further optimizes costs [1]. From a technical standpoint, it should be noted that dedicated ERCP devices are lacking for patients who require a pediatric duodenoscope due to its small diameter operative channel. This results in an augmented procedural complexity that needs to be managed at high-volume referral centers. Available data suggest that pediatric ERCP outcomes are comparable with those of adults, with similar or slightly higher adverse event rates [2,3,4,5]. This study reviews almost 20 years of pediatric ERCP practice at our center and focuses particularly on the subgroup weighing less than 10 kg.

## 2. Materials and Methods

All consecutive ERCPs performed at ISMETT from 2005 to 2023 on patients younger than 18 years old were identified through the electronic endoscopy database (Endobase, Olympus Corporation, Tokyo, Japan). Clinical data were retrieved from hospital medical records and collected in REDCap by two independent authors. The data included demographics (age, sex, weight), indications, interventions, outcomes (technical success), and adverse events.

Three experienced adult hepato-pancreato-biliary (HPB) endoscopists (MT, IT, DL) performed the procedures. In patients < 10 kg, a pediatric duodenoscope (PJF-160, Olympus Corporation, Tokyo, Japan) was used; otherwise, a standard duodenoscope (TJF-Q160V or TJF-Q180V, Olympus Corporation, Tokyo, Japan) was employed. A dedicated pediatric anesthesia team conducted the procedures in a dedicated fluoroscopy-equipped room under general anesthesia, with the patients in a supine position.

Technical success was defined as successful cannulation and opacification of the target duct for diagnostic ERCP or achievement of the intended therapeutic intervention for therapeutic ERCP. Adverse events (AEs) were assessed within two weeks post-procedure according to the American Society for Gastrointestinal Endoscopy (ASGE) lexicon and included post-ERCP pancreatitis (PEP), cholangitis, bleeding, and perforation.

Continuous variables were reported as means with standard deviations (SD) and categorical variables as frequencies with percentages. Because the number of ERCP procedures per patient is a count bounded below at one and was right-skewed, it is not summarized as mean ± SD but expressed as the ratio between the total number of procedures and the total number of patients in each weight group. Subgroup analyses were conducted on first-time ERCPs, stratified by weight and indication (pancreatic vs. biliary). Statistical analysis was performed using Fisher’s exact test and R software (version 2026.01.0+392), with a significance level set at *p* < 0.05. Given the small subgroup sizes and the sparse event counts, odds ratios were accompanied by exact conditional confidence intervals and risk differences by Newcombe hybrid-score confidence intervals, both of which remain valid when a cell contains zero events. Where one arm had no events the odds ratio is not estimable and is reported as such; a Haldane–Anscombe continuity correction (+0.5 per cell) is given for reference only. All subgroup comparisons were exploratory and unadjusted for multiplicity. Study approval was obtained from the ISMETT-IRCCS Ethical Committee (IRRB/11/24).

## 3. Results

A total of 194 pediatric ERCPs were performed on 84 patients and were categorized into two groups based on body weight: <10 kg (*n* = 11 procedures and *n* = 8 patients) and ≥10 kg (*n* = 183 procedures and *n* = 76 patients) (Table 1). The mean age in the <10 kg group was 1.2 ± 0.4 years, while the ≥10 kg group had a mean age of 9.8 ± 4.3 years. The male-to-female ratio was 1.2 in the <10 kg group and 0.95 in the ≥10 kg group. Mean weight was 8.1 ± 1.87 kg and 33.7 ± 17.3 kg, respectively. Children < 10 kg underwent 11 procedures in total and those ≥10 kg 183, corresponding to 1.4 and 2.4 procedures per patient, respectively; since this count was right-skewed, it is expressed as a ratio rather than as a mean with standard deviation.

**Figure 1:**
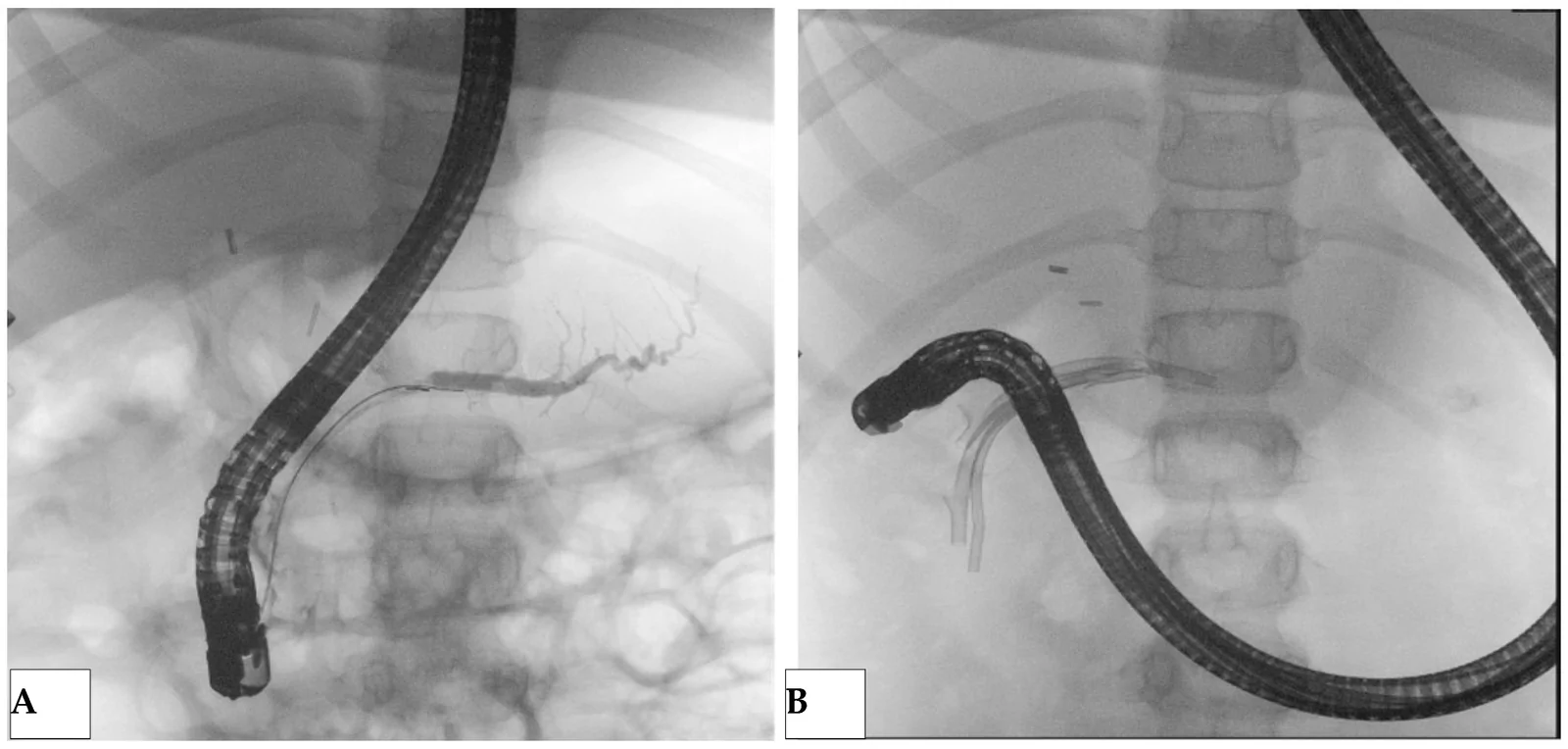
Chronic pancreatitis associated with incomplete pancreas divisum. (**A**) shows a stricture of the dorsal pancreatic duct in the pancreatic head with proximal dilation on pancreatogram. (**B**) shows a multistenting (side-by-side) approach of the dorsal duct through the minor papilla on pancreatogram.

**Figure 2:**
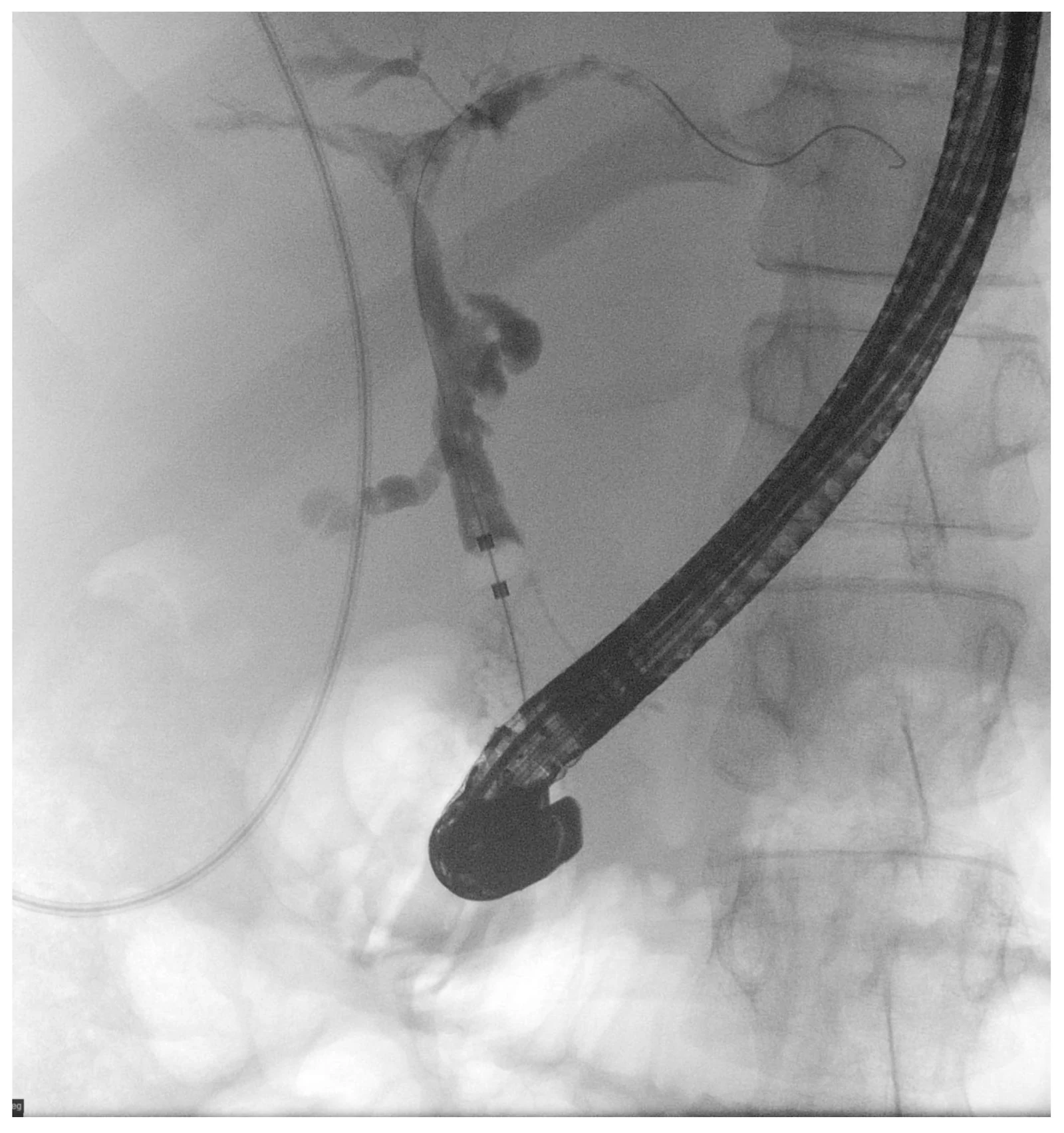
Cholangiogram showing a case of primary sclerosing cholangitis without dominant strictures.

**Figure 3:**
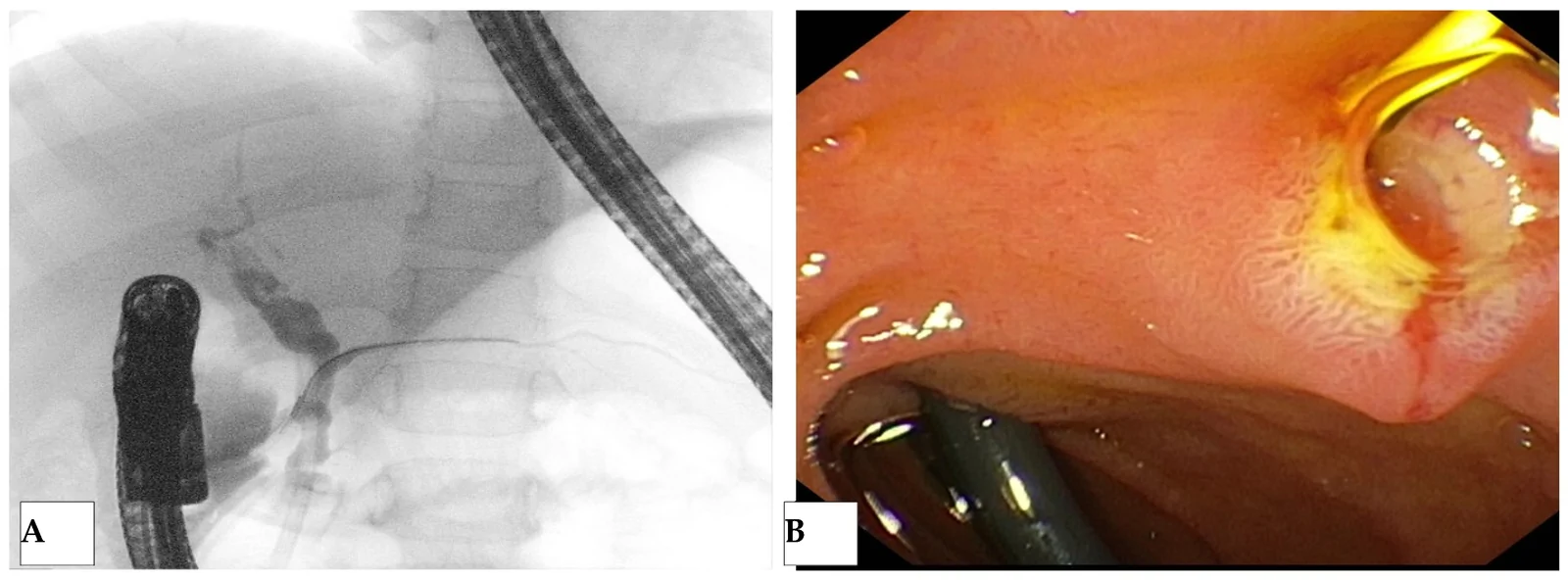
Incomplete pancreas divisum with dorsal pancreatic duct in communication with the choledocus. (**A**) shows the communication between the dorsal pancreatic duct and the choledocus on cholangio-pancreatogram; (**B**) shows the minor papilla with bile discharge.

Acute or chronic pancreatitis (Figure 1) were the most common indication for ERCP in both groups, representing 36% (*n* = 4) of cases in the <10 kg group and 36% (*n* = 66) in the ≥10 kg group (Table 2). In the <10 kg group, other relevant indications included biliary lithiasis (18%), primary sclerosing cholangitis (18%—Figure 2), and undefined biliary abnormality or obstruction (18%—Figure 3).

**Figure 4:**
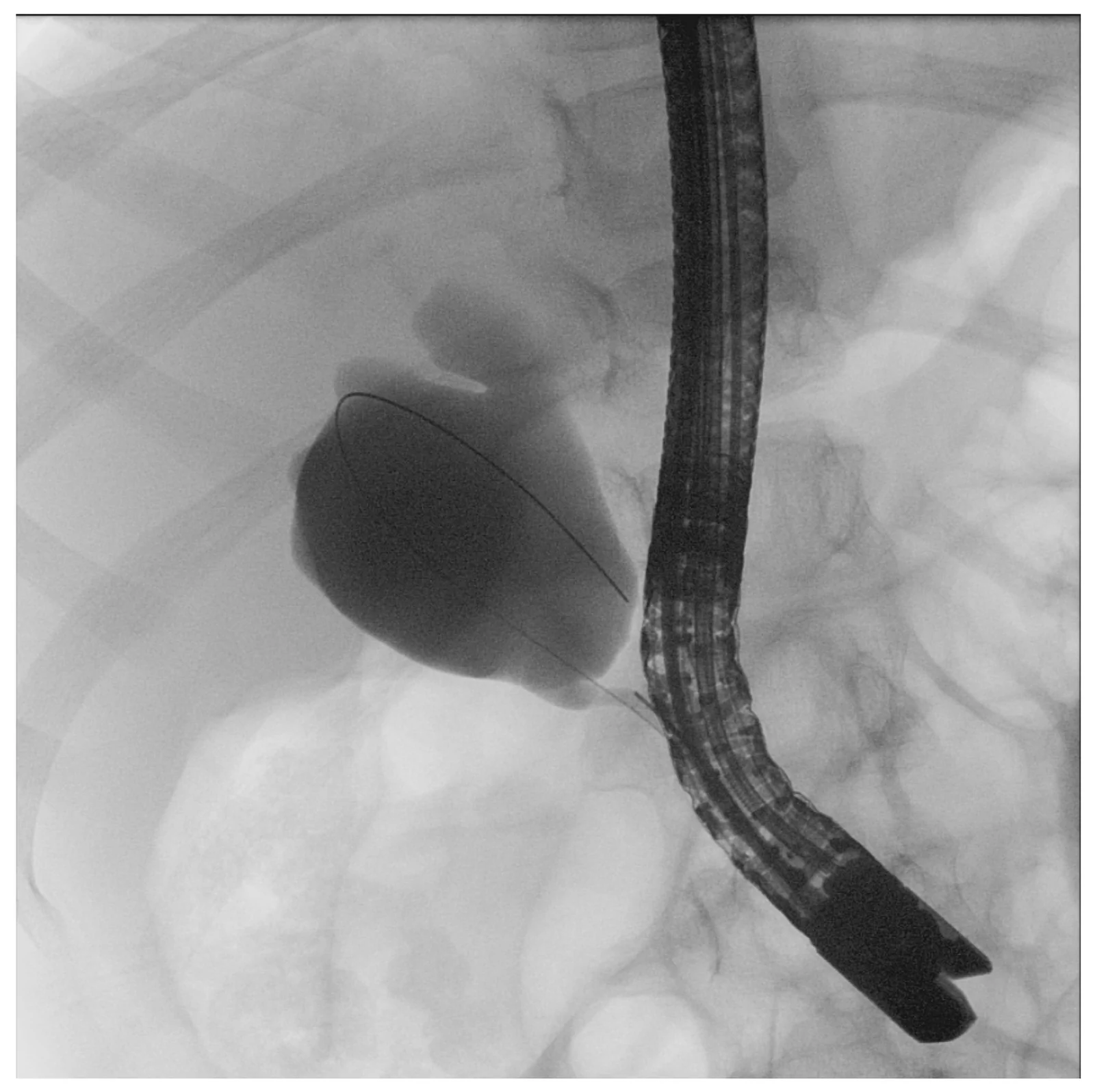
Cholangiogram showing a huge choledochal cyst.

In the ≥10 kg group, additional frequent indications were primary sclerosing cholangitis (14%), undefined biliary abnormality or obstruction (12%), post-transplant anastomotic biliary injury (10%), and biliary lithiasis (7%). Neoplastic biliary obstruction, choledochal cysts (Figure 4), and post-traumatic or post-surgical biliary leaks accounted for around 3% of cases each.

**Figure 5:**
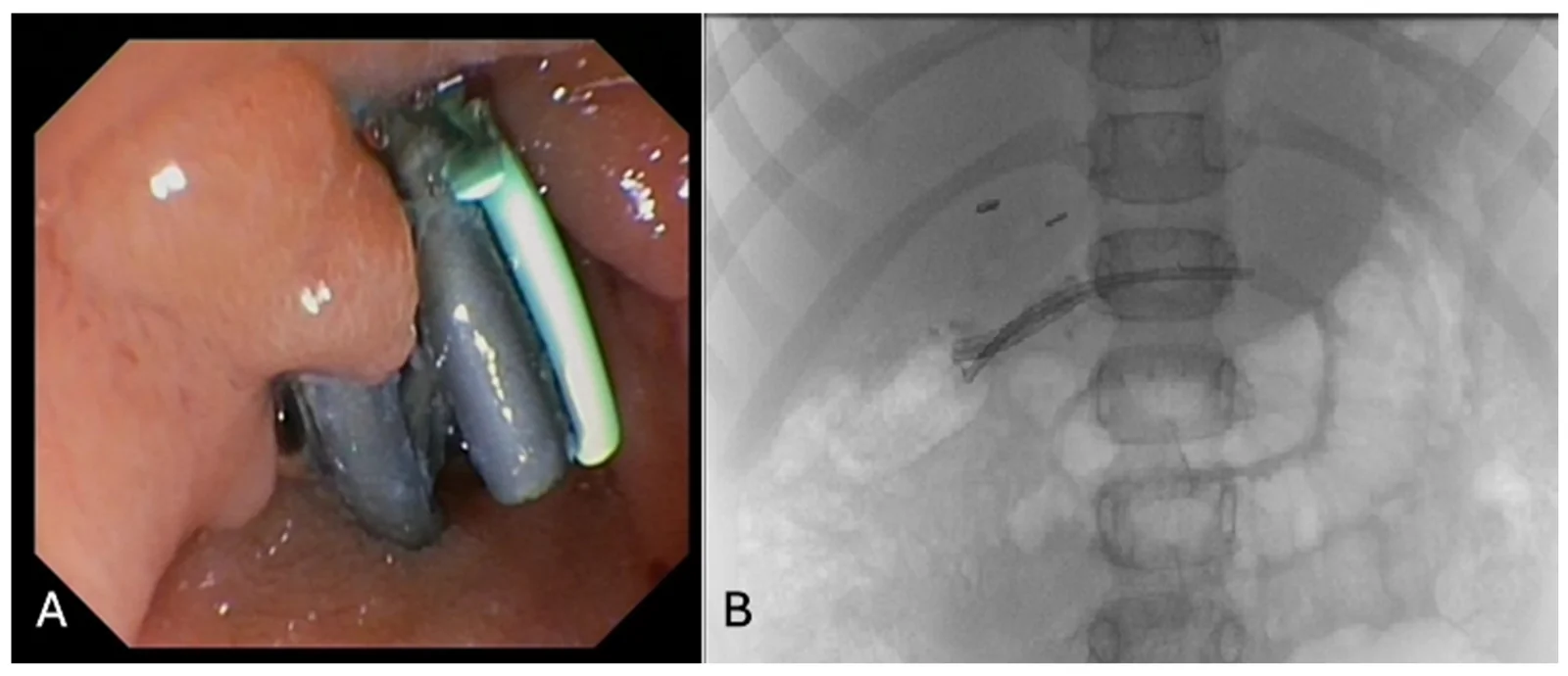
Multistenting (side-by-side) approach through the minor papilla. Endoscopic (**A**) and radiologic (**B**) views.

Regarding the procedural specifics, 100% of patients < 10 kg and 87% of those ≥10 kg underwent their first ERCP at our center (Table 3). Diagnostic procedures were performed on 36% of patients in the <10 kg group and 18% in the ≥10 kg group. Sphincterotomy was performed in 36% and 26% of cases, respectively. Stent placement was more frequent in the ≥10 kg group (45%) compared with the <10 kg group (27%). Biliary stone extraction was observed in 27% of children < 10 kg and 25% of those ≥10 kg. Other procedures, such as minor papilla cannulation (17%), pancreatic multistenting (7%—Figure 5), and stent removal (28%), were performed exclusively in the ≥10 kg group. Technical success was achieved in 91% of cases in the <10 kg group and 96% in the ≥10 kg group.

Adverse events occurred only in the ≥10 kg group and were reported in 7% of cases (Table 4). Specifically, PEP was the most frequent complication, occurring in 12 patients (6%). Bleeding and perforation were reported in 1 patient each (0.5%). No cases of cholangitis were recorded in either group. Notably, no adverse events were reported among patients weighing less than 10 kg.

All patients were admitted to the post-anesthesia care unit for recovery post-procedure, and none required intensive care unit admission. A subgroup analysis was performed on patients undergoing their first ERCP (Table 5). In this subgroup, 8 patients weighed <10 kg and 66 weighed ≥10 kg. Adverse events occurred in 0% of the <10 kg group and in 9% of the ≥10 kg group, with a risk difference of –9.09% (95% CI: –18.4 to +23.7); the odds ratio was not estimable because no events occurred in the <10 kg group, and Fisher’s exact test showed no significant difference (*p* = 1.000). Technical success was achieved in 87% and 94% of patients in the <10 kg and ≥10 kg groups, respectively, with no statistically significant difference (OR: 0.45; 95% CI: 0.04–4.63; *p* = 0.445).

When stratified by indication, patients with pancreatic indications (*n* = 10) experienced a higher rate of adverse events (30%) compared to those with biliary indications (4.7%; *n* = 64), with an odds ratio of 8.28 (exact 95% CI: 0.93–74.90; Fisher’s exact *p* = 0.029). Given that this estimate rests on three events per group, the interval is very wide and this association should be regarded as hypothesis-generating rather than definitive (Table 6). Technical success was slightly lower in the pancreatic group (80%) than in the biliary group (95.3%), but this difference was not statistically significant (OR: 0.20; 95% CI: 0.03–1.36; *p* = 0.132).

## 4. Discussion

In this study, we retrospectively analyzed the key characteristics of pediatric patients undergoing ERCP at our institution over 18 years of activity. To reduce heterogeneity, we stratified the cohort by weight, using a cutoff of 10 kg—the standard threshold at our center for choosing between pediatric and adult duodenoscopes and roughly corresponding to the “infant” versus “child” classification. In fact, anatomical cricopharyngeal characteristics also need to be considered in the choice of a standard vs. pediatric duodenoscope, even for patients weighing more than 10 kg and especially when they are affected by associated syndromes.

A notable finding was that pediatric patients weighing ≥ 10 kg underwent multiple ERCPs (2.4 procedures per patient), slightly higher than the number previously reported in other studies [2,5,6]. While Lorio et al. [7] reported that ERCPs in younger pediatric patients tended to be more complex and required repetition more frequently, our data indicate that, in our cohort, patients weighing less than 10 kg underwent fewer procedures on average than those weighing ≥ 10 kg. Pancreatitis emerged as the leading indication across both subgroups, differing from previous studies [2,5]. Diagnostic ERCP was notably more frequent in infants (36%) than in older children (18%), consistent with prior reports [5]. In fact, ERCP plays a key role in infants selected for surgery for whom non-invasive diagnostic workups, such as ultrasound and magnetic resonance cholangiopancreatography (MRCP), are inconclusive. Sphincterotomy, including minor papilla sphincterotomy, was performed in 36% of <10 kg and 26% of ≥10 kg patients. Multistenting procedures, both biliary and pancreatic, were performed exclusively on patients ≥ 10 kg (7% each), marking the first study to report such an approach in pediatric ERCP. Subgroup analysis focusing on initial ERCPs suggested a higher rate of adverse events for pancreatic than for biliary indications (30% vs. 4.7%; OR 8.28, exact 95% CI 0.93–74.90; *p* = 0.029), although the small number of events makes this estimate imprecise. Notably, no cases of PEP were reported among infants, an observation consistent with other studies [5,8] and one that highlights a potential area for further investigation.

Finally, a comment should be made regarding the general care of these patients. All procedures were performed under general anesthesia by a specialized pediatric liver transplantation team available at our facility. The choice of general anesthesia as the standard of care was made to ensure the maximal safety for airway management; indeed, the effectiveness of this approach with short-term drug use was confirmed by the need to stay only in the recovery room with no admissions to the intensive care unit.

A substantial limitation of this study is the absence of data on radiation exposure. Fluoroscopy time and dose–area product were not systematically recorded in the endoscopy and radiology archives over the whole study period, and they could not be reconstructed retrospectively for the earlier years of the cohort; we therefore cannot report cumulative exposure, which is a relevant omission in a pediatric fluoroscopy-guided series. This is a crucial issue since children are more sensitive to radiation effects [9]. They have a longer lifespan during which radiation-induced complications, such as cancer, can arise, and they are susceptible to receiving excessively high doses if fluoroscopy settings are not appropriately adapted to their smaller body size [1]. A further limitation concerns the statistical fragility of the subgroup analyses: the <10 kg subgroup comprised only eight first procedures with no adverse events, so the corresponding odds ratio is not estimable, and the pancreatic-versus-biliary comparison rests on three events in each group. Confidence intervals are therefore wide, a single additional event would materially change the estimates, and no adjustment for multiple comparisons was applied; these findings should be interpreted as hypothesis-generating and require confirmation in larger, ideally multicenter, cohorts. The absence of adverse events in infants should likewise be read as compatible with a low but non-zero risk rather than as evidence of no risk. However, the study benefits from a relatively large pediatric sample collected over nearly two decades at a high-volume, internationally recognized tertiary center, allowing for comparisons with the existing literature and the formulation of new research hypotheses.

## 5. Conclusions

In our single-center retrospective experience, ERCP was an effective diagnostic and therapeutic procedure in the pediatric population, with a low rate of peri-procedural adverse events. These findings apply to settings comparable to ours—high procedural volume, experienced biliopancreatic endoscopists, dedicated pediatric anesthesia and multidisciplinary support—and do not address long-term radiation-related risk, for which data were not available. Indications and technical strategies vary depending on patient characteristics, particularly weight and age. Pancreatic indications appeared to carry a higher risk of adverse events, a finding that warrants confirmation in larger cohorts.

## Figures and Tables

**Table 1:** Baseline characteristics—data refer to the total number of procedures.

	<10 Kg Mean (±SD) or n°	≥10 Kg Mean (±SD) or n°
Total patients	8	76
Total procedures	11	183
Mean age	1.2 (±0.4)	9.8 (±4.3)
Male:Female ratio	1.2	0.95
Mean weight	8.1 (±1.87)	33.7 (±17.3)
Mean n° of procedures per patient	1.4	2.4

**Table 2:** Indications—data refer to the total number of procedures.

	<10 Kg, n° (%)	≥10 Kg, n° (%)
Acute or chronic pancreatitis	4 (36)	66 (36)
Primary sclerosing cholangitis	2 (18)	26 (14)
Biliary lithiasis	2 (18)	13 (7)
Undefined biliary abnormality or obstruction	2 (18)	20 (12)
Neoplastic biliary obstruction	1 (9)	6 (3)
Post-transplant anastomotic biliary injury (stricture or leak)	0	18 (10)
Post-traumatic pancreatic duct injury (stricture or leak)	0	17 (9)
Post-surgical biliary leak	0	6 (3)
Choledochal cyst	0	6 (3)
Post-traumatic biliary leak	0	5 (3)

**Table 3:** Interventions—data refer to the total number of procedures.

	<10 Kg, n° (%)	≥10 Kg, n° (%)
First ERCP	8 (73)	66 (36)
Diagnostic procedures	4 (36)	33 (18)
Sphincterotomy (biliary or pancreatic—including minor papilla)	4 (36)	47 (26)
Precut (biliary or pancreatic—including minor papilla)	1 (9)	7 (4)
Papilla dilation (biliary or pancreatic—including minor papilla)	3 (27)	15 (8)
Minor papilla cannulation	0	32 (17)
Biliary anastomosis dilation	0	2 (1)
Biliary stone extraction	3 (27)	25 (14)
Stent placement	3 (27)	82 (45)
Biliary “multistenting approach”	0	13 (7)
Pancreatic “multistenting approach”	0	12 (7)
Stent removal	0	52 (28)
Technical success	10 (91)	175 (96)

**Table 4:** Adverse events—data refer to the total number of procedures.

	<10 Kg n° (%)	≥10 Kg n° (%)
Bleeding	0	1 (0.5)
Perforation	0	1 (0.5)
Post-ERCP pancreatitis	0	12 (6)
Cholangitis	0	0

**Table 5:** Subgroup analysis based on weight—first procedure.

	<10 Kg, n° (%)	≥10 Kg, n° (%)	RD (95% CI)	OR (95% CI)	*p*-Value
Total	8	66	-	-	-
Adverse event rates	0	6 (9)	−9.09% (−18.4/23.7)	Not estimable ^†^	1.000
Technical success	7 (87)	62 (94)	–6.44% (−30.07/17.19%)	0.45 (0.04/4.63)	0.445

^†^ Odds ratio not estimable because no adverse events occurred in the <10 kg group (zero cell). With the Haldane–Anscombe continuity correction (+0.5 per cell), the odds ratio would be 0.55 (95% CI 0.03–9.65).

**Table 6:** Subgroup analysis based on procedure type—first procedure.

	Pancreatic Procedures, n° (%)	Biliary Procedures, n° (%)	RD (95% CI)	OR (95% CI)	*p*-Value
Total	10	64	-	-	-
Adverse event rates	3 (30)	3 (4.7)	25.3% (4.4/55.8%)	8.28 (0.93/74.90)	0.029
Technical success	8 (80)	61 (95.3)	−15.3% (−40.6%/10.0%)	0.20 (0.03/1.36)	0.132

## Data Availability

The original contributions presented in this study are included in the article. Further inquiries can be directed to the corresponding author.

## Abbreviations

The following abbreviations are used in this manuscript:

ERCP	Endoscopic retrograde cholangiopancreatography
AEs	Adverse Events
PEP	Post-ERCP Pancreatitis
ASGE	American Society for Gastrointestinal Endoscopy
IRRB	Institutional Review Board
HPB	Hepato-Pancreato-Biliary
MRCP	Magnetic Resonance Cholangiopancreatography

## References

[1] Baron T.H., Kozarek R.A., Carr-Locke D.L. (2019). ERCP.

[2] Enestvedt B.K., Tofani C., Lee D.Y., Abraham M., Shah P., Chandrasekhara V., Ginsberg G.G., Long W., Ahmad N., Jaffe D.L. (2013). Endoscopic Retrograde Cholangiopancreatography in the Pediatric Population Is Safe and Efficacious. J. Pediatr. Gastroenterol. Nutr..

[3] Giefer M.J., Kozarek R.A. (2015). Technical outcomes and complications of pediatric ERCP. Surg. Endosc..

[4] Hiramatsu T., Itoh A., Kawashima H., Ohno E., Itoh Y., Sugimoto H., Sumi H., Funasaka K., Nakamura M., Miyahara R. (2015). Usefulness and safety of endoscopic retrograde cholangiopancreatography in children with pancreaticobiliary maljunction. J. Pediatr. Surg..

[5] Åvitsland T.L., Aabakken L. (2021). Endoscopic retrograde cholangiopancreatography in infants and children. Endosc. Int. Open.

[6] Varadarajulu S., Wilcox C.M., Hawes R.H., Cotton P.B. (2004). Technical outcomes and complications of ERCP in children. Gastrointest. Endosc..

[7] Lorio E., Moreau C., Hernandez B., Rabbani T., Michaud K., Hachem J., Aggarwal P., Stolow E., Brown L., Michalek J.E. (2023). Pediatric ERCP: Factors for Success and Complication-A 17-Year, Multisite Experience. J. Pediatr. Gastroenterol. Nutr..

[8] Shanmugam N.P., Harrison P.M., Devlin J., Peddu P., Knisely A., Davenport M., Hadžić N. (2009). Selective Use of Endoscopic Retrograde Cholangiopancreatography in the Diagnosis of Biliary Atresia in Infants Younger Than 100 Days. J. Pediatr. Gastroenterol. Nutr..

[9] Dumonceau J.-M., Garcia-Fernandez F., Verdun F., Carinou E., Donadille L., Damilakis J., Mouzas I., Paraskeva K., Ruiz-Lopez N., Struelens L. (2012). Radiation protection in digestive endoscopy: European Society of Digestive Endoscopy (ESGE) Guideline. Endoscopy.

